# Zebrafish P54 RNA helicases are cytoplasmic granule residents that are required for development and stress resilience

**DOI:** 10.1242/bio.015826

**Published:** 2016-08-03

**Authors:** Cecilia Zampedri, Maryana Tinoco-Cuellar, Samantha Carrillo-Rosas, Abigail Diaz-Tellez, Jose Luis Ramos-Balderas, Francisco Pelegri, Ernesto Maldonado

**Affiliations:** 1 EvoDevo Laboratory, Unidad de Sistemas Arrecifales, Instituto de Ciencias del Mar y Limnología, Universidad Nacional Autónoma de México, Puerto Morelos, Quintana Roo, México, 77580; 2 Departamento de Biología Celular y del Desarrollo, Instituto de Fisiología Celular, Universidad Nacional Autónoma de México, D.F. México, México, 04510; 3 Laboratory of Genetics, University of Wisconsin-Madison, Wisconsin 53706, USA

**Keywords:** P54 RNA helicase, P-bodies, Stress granules, Zebrafish

## Abstract

Stress granules are cytoplasmic foci that directly respond to the protein synthesis status of the cell. Various environmental insults, such as oxidative stress or extreme heat, block protein synthesis; consequently, mRNA will stall in translation, and stress granules will immediately form and become enriched with mRNAs. P54 DEAD box RNA helicases are components of RNA granules such as P-bodies and stress granules. We studied the expression, in cytoplasmic foci, of both zebrafish P54 RNA helicases (P54a and P54b) during development and found that they are expressed in cytoplasmic granules under both normal conditions and stress conditions. In zebrafish embryos exposed to heat shock, some proportion of P54a and P54b helicases move to larger granules that exhibit the properties of genuine stress granules. Knockdown of P54a and/or P54b in zebrafish embryos produces developmental abnormalities restricted to the posterior trunk; further, these embryos do not form stress granules, and their survival upon exposure to heat-shock conditions is compromised. Our observations fit the model that cells lacking stress granules have no resilience or ability to recover once the stress has ended, indicating that stress granules play an essential role in the way organisms adapt to a changing environment.

## Abstract (precis)

**Summary:** Stress granules are formed by mRNAs stalled in translation during stress conditions. P54 RNA helicases from zebrafish reside in cytoplasmic granules and are essential for heat-shock resilience.

## INTRODUCTION

In eukaryotic cells, mRNA regulation is carried out in multiple parts of the cell and during multiple stages of the cell cycle. Important reservoirs for mRNAs are the RNA granules, which include P-bodies (Oh et al., 2013; Parker and Sheth, 2007), stress granules (Anderson and Kedersha, 2008; Kedersha et al., 2002; Sahoo et al., 2012), germ granules (in germ cells) (Updike and Strome, 2010), and other types of RNA-containing cytoplasmic foci. This classification of RNA granules is based on their composition, size and cell of origin. The mechanism leading to the formation of RNA granules remains a matter of debate (Ramaswami et al., 2013; Weber and Brangwynne, 2012). A remarkable aspect of RNA-containing granules is the absence of encapsulating membranes which leaves RNA and associated RNA-binding proteins free to shuttle in and out of granules in a dynamic equilibrium, rendering such aggregates unstable by nature (Buchan and Parker, 2009). For this reason, the isolation of RNA-containing granules from cells and their further characterization *in vitro* remain issues that have proven difficult to address. The different classes of RNA granules share common features. They possess mRNAs in a repressed state that may re-initate translation in response to specific signals (Bhattacharyya et al., 2006; Brengues et al., 2005; Nagamori et al., 2011). Further, they exhibit dynamic interactions with one another, such as docking, fusion, or apparent maturation from one granule type to the next (Hoyle et al., 2007; Kedersha et al., 2005). Meanwhile, RNA granules share certain components, such as RNA-binding proteins and certain mRNAs (Buchan and Parker, 2009), and frequently, some components shuffle from one type of granule to another granule type as cellular conditions change (Buchan et al., 2008; Kedersha et al., 2005; Mollet et al., 2008).

One of the most-studied shared components of different types of granules is the DEAD-box P54/RCK RNA helicase. This protein is a member of a helicase DDX6 subfamily, conserved in invertebrates and vertebrates, with homologues in human (RCK/P54), mouse (P54), *Xenopus* (Xp54), *Drosophila* (Me31B), *Caenorhabditis* *elegans* (Cgh-1), *Planaria* (DjCBC-1), and *Saccharomyces* *cerevisiae* (Dhh1) (Navarro and Blackwell, 2005; Navarro et al., 2001; Rajyaguru and Parker, 2009; Weston and Sommerville, 2006; Yoshida-Kashikawa et al., 2007). In mammalian cells, depletion of P54/RCK protein leads to the disappearance of P-bodies and prevents their *de novo* assembly in response to triggers such as arsenite, which means that P54/RCK is central to P-body assembly (Serman et al., 2007).

It also has been reported that P54/RCK interacts with P-bodies/decapping proteins (Bish et al., 2015) and with the RISC complex, which mediates translational silencing by miRNAs (Chu and Rana, 2006). Ddx6 also interacts with two stress granule proteins (GRAN1 and GRAN2), even under normal conditions, when visible mRNP structures are absent, suggesting that Ddx6 may be a key factor in modulating the contents of P-bodies and stress granules (Bish et al., 2015). Xp54 in *Xenopus* is known as a component of the CPEB repressor complex in oocytes (Ladomery et al., 1997; Minshall et al., 2001), and in *C. elegans*, the P54 homolog Cgh-1 (Navarro and Blackwell, 2005) promotes both mRNA stability in P-granules in oocytes and mRNA decay in somatic cell granules (Boag et al., 2008; Noble et al., 2008). In zebrafish (*Danio rerio*), stress granules were identified, while studying FUS (fused in sarcoma) abnormal cytoplasmic localization, by the use of antibodies against classic stress granules markers such as TIAL-1 (T-cell internal antigen like-1) (Bosco et al., 2010) and eIF3e (Acosta et al., 2014).

Stress granules are formed in response to various stressors, and their components include translation initiation factors (eIF3, eIF4A, eIF4E and eIF4G), stalled mRNAs (Anderson and Kedersha, 2008; Anderson et al., 2015), 40S ribosomal subunits, and RNA binding proteins (RBPs). Some examples for these RBPs are TIA1 (T-cell internal antigen 1), TIAL-1 (T-cell internal antigen like-1) (Kedersha et al., 2000), and G3BP1 (Ras-GTPase-activating protein SH3-domain binding protein 1) (Tourriere et al., 2003). These granules are in a dynamic equilibrium with polysomal mRNA; it was observed in photobleaching experiments that they respond directly to the translational status of the cell (Mollet et al., 2008). Global inhibition of protein synthesis induces coalescence of stress granule components; nevertheless, the molecular details of the aggregation process are not clearly understood.

In this work, we characterize P54/RCK homologs P54a and P54b in zebrafish, particularly their residence in cytoplasmic foci that are similar to P-bodies and to stress granules. Using a combination of heat-shock treatments and classical translation inhibitors (cycloheximide and puromycin) we found that P54 granules exhibit the properties of classical stress granules. This work also shows that P54 granules are essential for whole-organism development and resilience or the ability to recover after heat shock.

## RESULTS

### Zebrafish have two P54 DEAD box RNA helicases

**Fig. 1.**
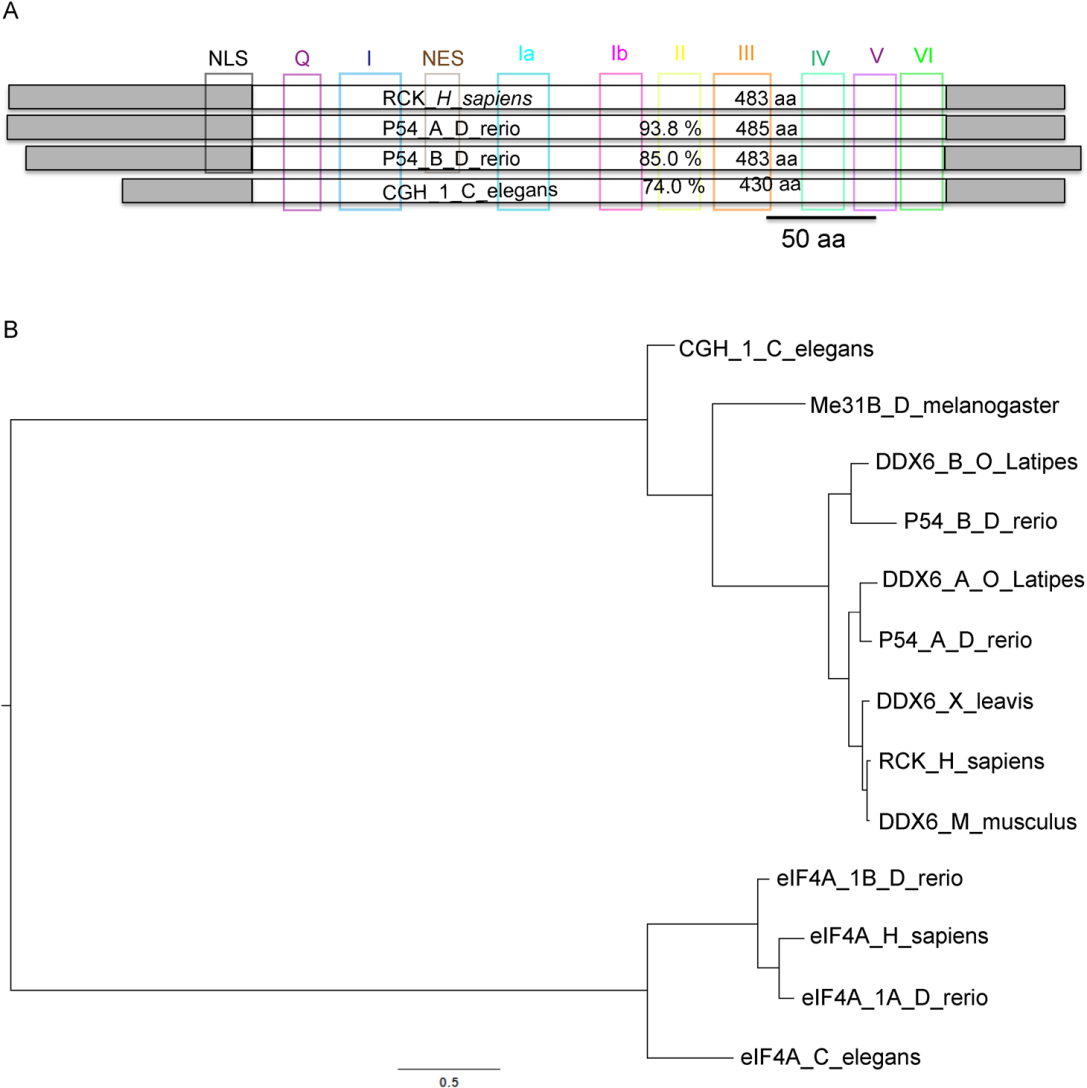
**Domain structure and evolutionary conservation of the P54 RNA helicases P54a and P54b from zebrafish.** (A) Conserved domains in P54 RNA helicases (NLS, Q, I, NES, Ia, Ib, II, III, IV, V and VI) are indicated in colored boxes. Zebrafish P54a and P54b proteins are compared with human RCK (Uniprot P26196) and CGH_1 from *C. elegans* (Wormbase C07H6.5) proteins. The central white boxes represent the conserved helicase region, and its percentage identity with RCK is shown. Gray boxes indicate C- and N-terminal variable regions. (B) Maximum likelihood tree of P54-related proteins. The scale indicates substitutions per site. The outgroup is represented by eIF4A RNA helicases from various organisms. CGH-1, Me31B, DDX6 and RCK are different names for the P54 RNA helicase. Abbreviations: *H. sapiens* (*Homo sapiens*), *D. rerio* (*Danio rerio*), *C. elegans* (*Caenorhabditis elegans*), *D. melanogaster* (*Drosophila melanogaster*), *O. latipes* (*Oryzias latipes* or medaka fish), *X. laevis* (*Xenopus laevis*), *M. musculus* (*Mus musculus*).

Human Rck/p54 and *Caenorhabditis elegans cgh-1* belong to a family of DEAD box RNA helicases, closely related to eIF4A that allows translation initiation by mRNA unwinding (Linder and Fuller-Pace, 2013). In the zebrafish (*Danio rerio*) genome, we found two open reading frames in the Ensembl databases; ENSDARP00000081816.5 (Chromosome 18:43.87 Mb) and ENSDARP00000129311.1 (Chromosome 16:25.76 Mb). According to protein sequence multiple alignment and phylogenetic analysis (Fig. 1) we found both genes to be orthologs of CGH-1 in *C. elegans*, Me31B in *Drosophila melanogaster*, DDX6 in *Xenopus laevis* and the human Rck/p54 family of DEAD box RNA helicases. We named them P54a and P54b, respectively. All conserved domains from this DEAD box protein family are also conserved in zebrafish P54a and P54b, including the ATP-binding domain I and RNA-binding motifs IV and V. The conserved NLS (nuclear localization signal) and NES (nuclear export signal) sequences, only found in P54 RNA helicases from vertebrates, were also found in zebrafish P54 proteins (Fig. 1A). In a phylogenetic tree of selected DEAD box RNA helicases, the eIF4A branch is clearly an outgroup from the P54/RCK/Cgh-1 branch (Fig. 1B). All known genomes from teleost fishes contain both P54a and P54b RNA helicases (data not shown); in zebrafish, the presence of duplicated genes is a common feature due to an ancient genome duplication during the evolution of ray-finned fish (Glasauer and Neuhauss, 2014). P54a appears to be more closely related to P54 from mammals than P54b (93.8% and 85% identity with the human ortholog, respectively).

### P54a and P54b are both expressed in cytoplasmic granules during zebrafish development

**Fig. 2.**
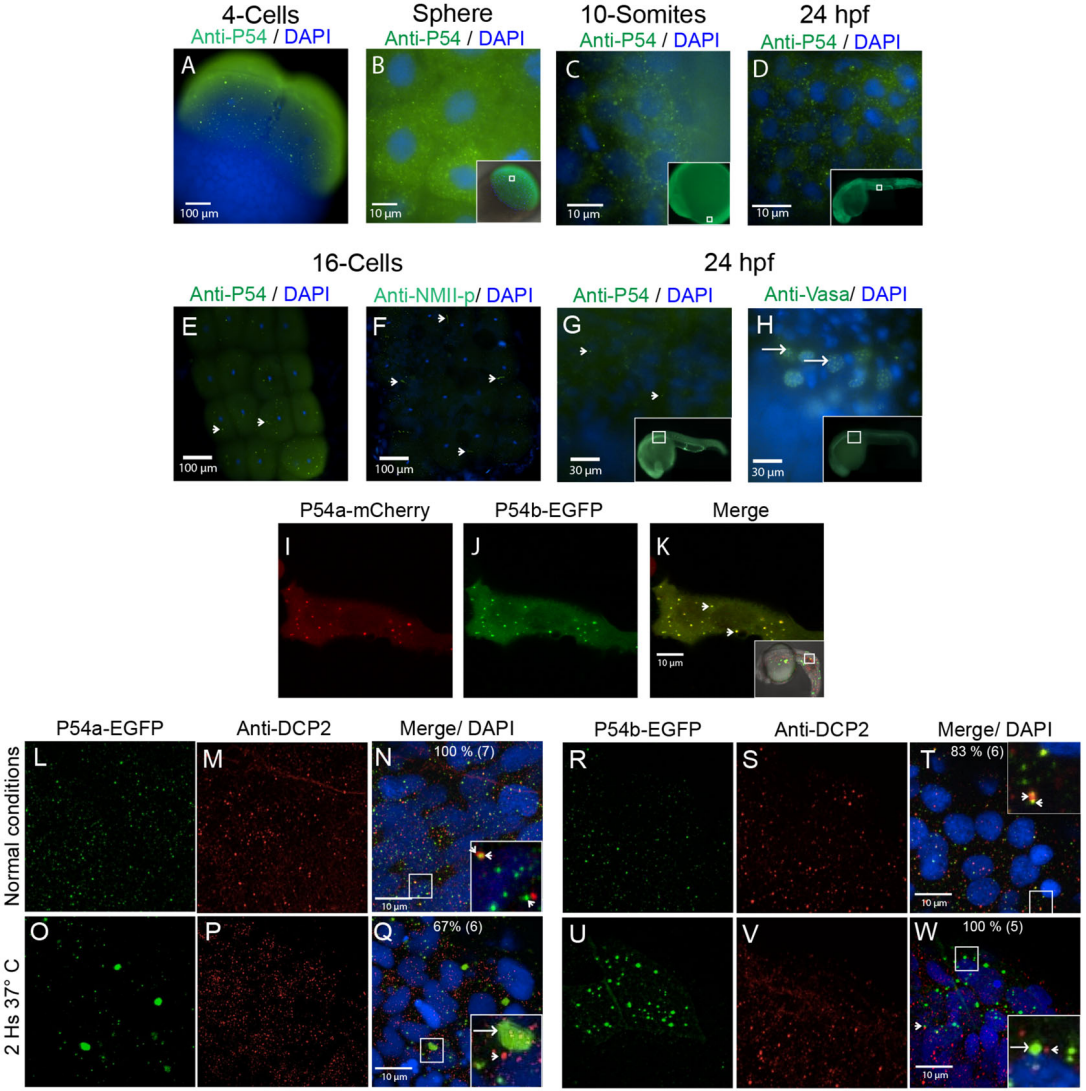
**P54 proteins are expressed in cytoplasmic granules during zebrafish development.** (A–E,G) Whole-mount immunostaining was performed with the anti-P54 antibody in WT zebrafish embryos at (A) the 4-cell stage, (B) the sphere stage, (C) the 10-somite stage, and (D,E,G) 24 hpf. In these embryos, P54 helicases were located in cytoplasmic granules. Immunostaining (green) and DAPI (blue) were visualized by epifluorescence microscopy. (E–H) P54-positive granules were different from germline granules, observed by immunostaining with anti-NMII-p (16-cell stage) or anti-Vasa (24 hpf), two known markers of germ granules. Arrowheads indicate P54-positive granules, and arrows indicate granules labeled by anti-NMII-p in germplasm or anti-Vasa in germ granules. (I–K) Live embryos at 24 hpf expressing the P54a-mCherry and P54b-EGFP fusion proteins that were also located in cytoplasmic granules. Arrows indicate cytoplasmic granules where P54a and P54b co-localize. (L–W) Expression of P54a-EGFP and P54b-EGFP fusion reporters and immunostaining with anti-Dcp2 (a marker for P-bodies) in 24 hpf embryos maintained at normal temperature (28.5°C) (L–N,R–T) or exposed for 2 h to heat-shock conditions (37°C) (O–Q,U–W). P54a and P54b fusion proteins co-localize with or are in close contact with Dcp2-labeled granules. Under heat-shock conditions, P54a-EGFP and P54b-EGFP were observed in larger granules that can be seen in close contact with smaller Dcp2-positive granules. Small granules, such as P-bodies, are labeled with arrowheads, and larger granules are indicated with arrows. For some images, a general view of the embryos is shown in the inset, where white boxes indicate the regions where the analysis was performed.

P54 DEAD box RNA helicases have been studied in several organisms and are usually found in cytoplasmic granules with RNA processing functions (Presnyak and Coller, 2013). We used an antibody against P54 proteins (see the Material and Methods section and Fig. S1) to locate the expression of P54 RNA helicases during different developmental stages in zebrafish. P54 was observed both in cytoplasmic granules and diffused in the cytoplasm, beginning very early in development, at the 4-cell stage [1 hour post-fertilization (hpf); Fig. 2A] and later at the sphere (4 hpf), 10-somites (10 hpf) stage and in 24 hpf embryos (Fig. 2B–D). Although both zebrafish P54 helicases have nuclear localization sequences, we did not observe any nuclear labeling. Because P54 has been found in germ granules in *C. elegans* (P-granules) and *Drosophila melanogaster* (Polar granules) (Nakamura et al., 2001; Navarro et al., 2001), we compared the expression of P54 with two known markers for zebrafish germ granules; phosphorylated non-muscle myosin (NMII-p) and Vasa. Unexpectedly the expression pattern of P54 at the 16-cell stage (1.5 hpf) did not resemble the labeling reported for NMII-p at the cell division furrows (Nair et al., 2013), neither resemble the typical 24 hpf germ granule anti-Vasa staining (Braat et al., 2000) (Fig. 2E–H). These results indicate that P54 RNA helicases may not associate with germ granules in zebrafish. However, we observed that P54 immunostaining during zebrafish development (Fig. 2A–H) resembles the labeling of cytoplasmic granules known as P-bodies, where P54 RNA helicases homologs are known to reside (Minshall et al., 2009; Navarro and Blackwell, 2005; Presnyak and Coller, 2013).

Our anti-P54 serum could not distinguish whether these putative P-bodies contain both P54a and P54b RNA helicases. In order to solve this problem, we made C-terminal mCherry and EGFP fusion proteins with both P54a and P54b, respectively, and expressed these fusion proteins in zebrafish embryos. Both P54a-mCherry and P54b-EGFP fusion proteins were found to co-localize in cytoplasmic granules (Fig. 2I–K); some labeling was also observed diffused in the cytoplasm. This is a similar localization pattern to the one observed using the anti-P54 serum (Fig. 2D; Fig. S2C). In order to determine whether these cytoplasmic granules correspond to P-bodies, embryos expressing either P54a-EGFP or P54b-EGFP were immunostained using a bona fide marker for P-bodies, the anti-Dcp2 antibody, that binds to the mRNA deccaping protein-2. We observed similar punctuated pattern for P54a/P54b and Dcp2 but unexpectedly very few of them show co-localization (Fig. 2L–N,R–T). These data suggest that some P54a and P54b foci could correspond to P-bodies even though they are located in different granules.

While P-bodies are constitutive cytoplasmic granules required for RNA storage and decay, some other cytoplasmic granules only appear under stress conditions (Anderson et al., 2015). These are known as stress granules, and it has been shown that P54 RNA helicases are also components of stress granules in other organisms (Wilczynska et al., 2005). Zebrafish embryos expressing P54a-EGFP or P54b-EGFP were exposed to heat-shock conditions and immunostained with anti-Dcp2. As expected, P-bodies (Dcp2 positive) did not change in size with the heat-shock treatment, but, interestingly, P54a and P54b fusion proteins aggregated in larger foci during this stress condition (Fig. 2O–Q,U–W). These results suggest that zebrafish have large, heat-shock-dependent P54a and P54b cytoplasmic granules that resemble stress granules. It is important to mention that most of our observations were carried out at the trunk anterior regions of the 24 hpf embryo, and were from muscle cells or epithelial cells from the skin (Fig. S2).

### P54 RNA helicases from zebrafish are components of stress granules

**Fig. 3.**
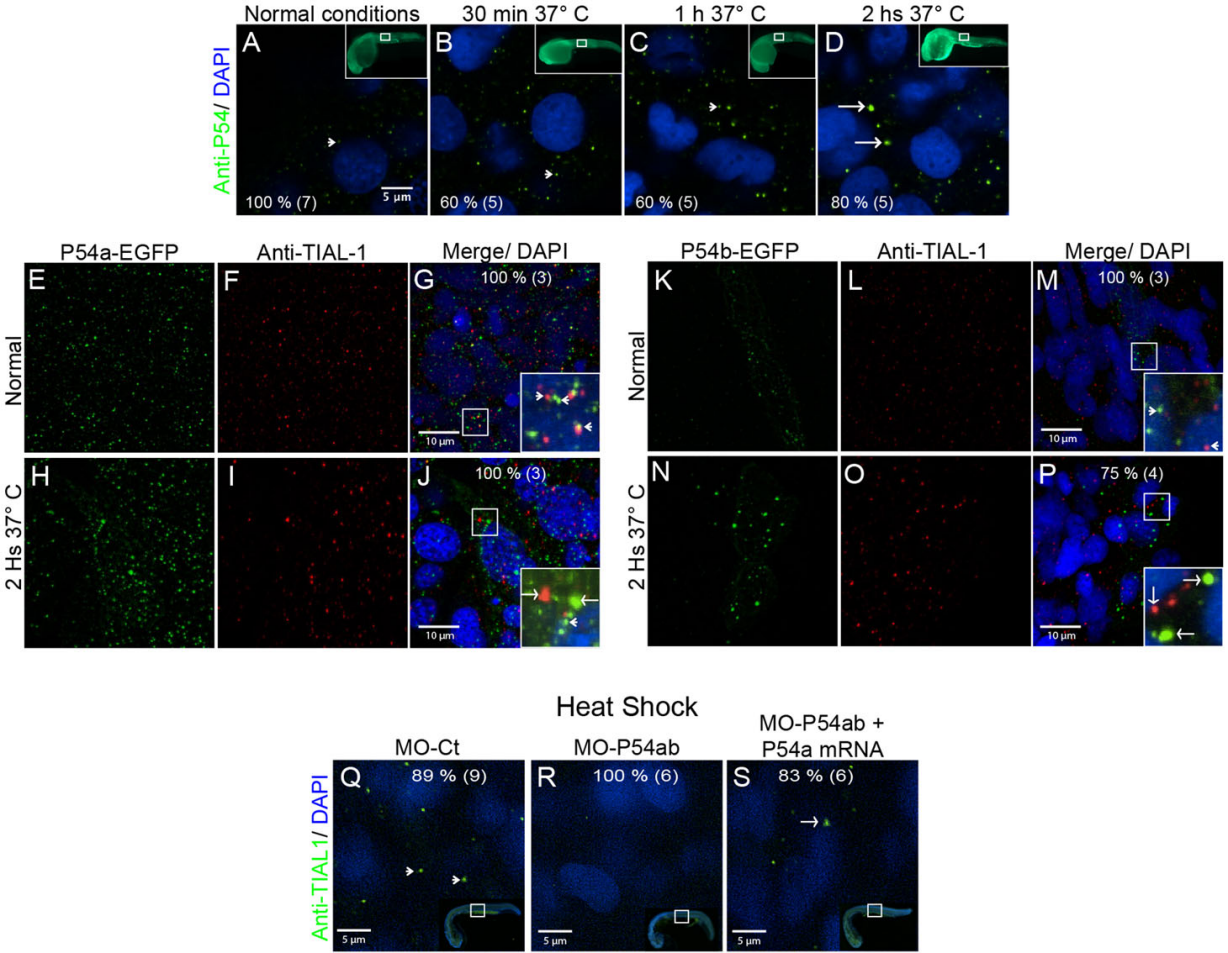
**Heat-shock induced the association of P54a and P54b RNA helicases with large cytoplasmic granules.** (A,E–G,K–M,Q) Wild-type 24 hpf zebrafish embryos were maintained under normal conditions at 28.5°C or (B–D,H–J,N–P,R,S) under heat-shock conditions at 37°C. (A–D) Anti-P54 immunostaining in embryos subjected to heat-shock for 30 min, 1 h and 2 h. Nuclei are counterstained with DAPI. The top right insets are general views, and the amplified regions are indicated in white boxes. (E–J) Expression of the P54a-EGFP in combination with TIAL-1 immunostaining (as a stress granule marker). In the inset, some P54a-expressing granules co-localize with or are in close contact with TIAL-1-positive granules. (K–P) P54b-EGFP expression in small (at 28.5°C) or large (at 37°C) granules, combined with immunostaining with anti-TIAL-1. The insets show both P54b-expressing granules and TIAL-1-positive granules. Arrowheads point to small granules, such as P-bodies, and arrows indicate larger granules, such as TIAL-1-positive (red) stress granules. (Q–S) Knockdown of P54a and P54b expression by splice-blocking morpholinos prevents the formation of stress granules labeled by anti-TIAL-1. For each panel, the percentage of embryos showing the same pattern is indicated, and the total number of fish embryos tested is shown in parentheses. White arrowheads point to small granules (putative P-bodies) and white arrows to large granules (putative stress granules).

To learn more about P54 granules dynamics during heat shock, we exposed 24 hpf embryos grown at 28.5°C, to 37°C for 30 min, 1 h or 2 h, followed by fixation and immunostaining using the anti-P54 specific serum. Under normal conditions, P54 was observed in small, discrete foci in the cytoplasm of most cells in the embryos (Fig. 3A). It was only after heat-shock that P54 RNA helicases were observed in larger granules, and the ratio of small to larger granules was increased as heat shock conditions were extended (Fig. 3B–D). In normal conditions, the average diameter measured for P54a-EGFP and P54b-EGFP cytoplasmic granules was 0.3 and 0.29 μm, respectively. By contrast, in heat shock conditions, P54a-EGFP and P54b-EGFP cytoplasmic granules have an average diameter of 1.6 and 1.0 μm respectively (Fig. S3). It is interesting that P54a-EGFP granules are larger than P54b-EGFP cytoplasmic granules, under heat shock conditions.

TIAL-1 is classic marker for stress granules in mammals (Kedersha et al., 1999; Moutaoufik et al., 2014). Therefore, to compare P54 heat-shock-induced cytoplasmic granules with TIAL-1-labeled stress granules we combined anti-TIAL-1 immunostaining and the expression of P54 fusion proteins. Specifically, we used 24 hpf embryos expressing either P54a-EGFP or P54b-EGFP reporters that also were immunostained with the anti-TIAL-1 antibody. We found that recombinant P54a-EGFP and P54b-EGFP reporters are localized in larger cytoplasmic granules induced by heat-shock (Fig. 3E,H,K,L; Fig. S3). As expected, the same dynamics was also observed in stress granules labeled with the anti-TIAL-1 antibody (Fig. 3F,I,L,O). However, we did not observe co-localization between P54a/P54b fusion proteins and anti-TIAL-1-labeled stress granules. Interestingly we did observe P54a-EGFP and P54b-EGFP granules adjacent to stress granules (Fig. 3G,J,M,P). It is not unusual to find cytoplasmic RNA granules of different types (such as stress granules and P-bodies) in close contact with each other; this has been seen as an indication of active flow of RNAs and proteins between them (Anderson and Kedersha, 2008).

**Fig. 4.**
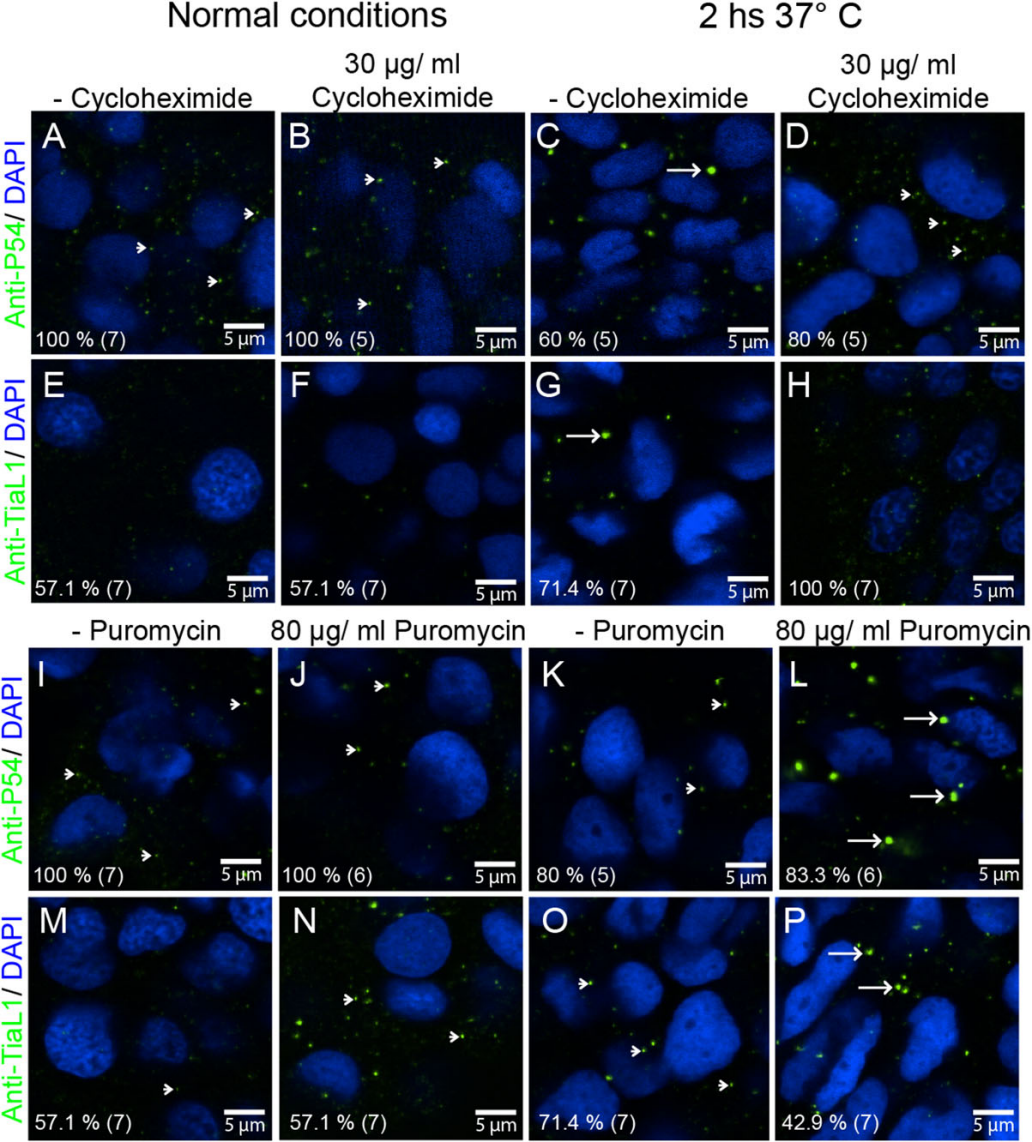
**P54 RNA helicase cytoplasmic granules exhibit the properties of stress granules.** (A–D,I–L) Anti-P54 immunostaining in 24 hpf WT zebrafish embryos. (E–H,M–P) Anti-TIAL-1 immunostaining as a marker for stress granules in 24 hpf WT embryos. (A–H) Some embryos were treated for 2 h with cycloheximide to block the formation of stress granules. (I–P) Puromycin was added (also for 2 h) to some of the embryos to enhance the assembly of stress granules. (A,B,E,F,I,J,M,N) Fish embryos were maintained at 28.5°C during cycloheximide and puromycin treatments or (C,D,G,H,K,L,O,P) at 37°C. After treatments and immunostaining, fluorescence signals were visualized by confocal microscopy. Arrowheads point to smaller granules (P-body size), and arrows point to larger stress granules. Anti-P54 and anti-TIAL-1 antibodies are both shown in green and DAPI nuclei staining in blue. At the bottom left of each image, the percentage of embryos showing the same immune reaction pattern is indicated, with the total number of embryos treated in parentheses.

Next, we reasoned that if P54 RNA helicases are essential for stress granule formation, knocking down their expression may affect the induction of heat shock stress granules. We knocked down the expression of both P54a and P54b in zebrafish by microinjection of splice-blocking morpholinos (see the Materials and Methods section and Fig. S1). This blocked the formation of TIAL-1-labeled stress granules under heat shock conditions, but the expression of those granules was rescued by the co-injection of ‘*in vitro*’ synthesized mature *p54* mRNA (*p54*-mRNA; Fig. 3Q–S). The absence of stress granules has also been observed upon the treatment of HeLa cells with the protein synthesis inhibitor cycloheximide (Wilczynska et al., 2005), which blocks translation elongation, thereby leading to the loss of stress granules by mRNA stabilization into polysomes. To find out more about the nature of TIAL-1 and P54 foci, 24 hpf zebrafish embryos were treated for 2 h with cycloheximide and simultaneously placed under heat shock conditions. Cycloheximide treatment did not affect P54 or TIAL-1 foci under normal temperature conditions (Fig. 4A,B,E,F), however we observed a reduction in the number of P54 large granules, and failed to observe any TIAL-1 labeled stress granules in embryos treated with cycloheximide and heat shock (Fig. 4A–H). Opposite to the cycloheximide effect, the protein synthesis inhibitor puromycin, which blocks translation initiation, has been reported to boost the assembly of stress granules by releasing mRNAs from polysomes (Wilczynska et al., 2005). Zebrafish embryos treated simultaneously with puromycin and heat shock showed even larger granules detected by both the P54 anti-serum and the TIAL-1 antibody (Fig. 4I–P). These experiments suggest that P54 helicases in zebrafish are components of granules that are morphologically and functionally similar to stress granules.

### Developmental effects of blocking the expression of P54 RNA helicases in zebrafish

**Fig. 5.**
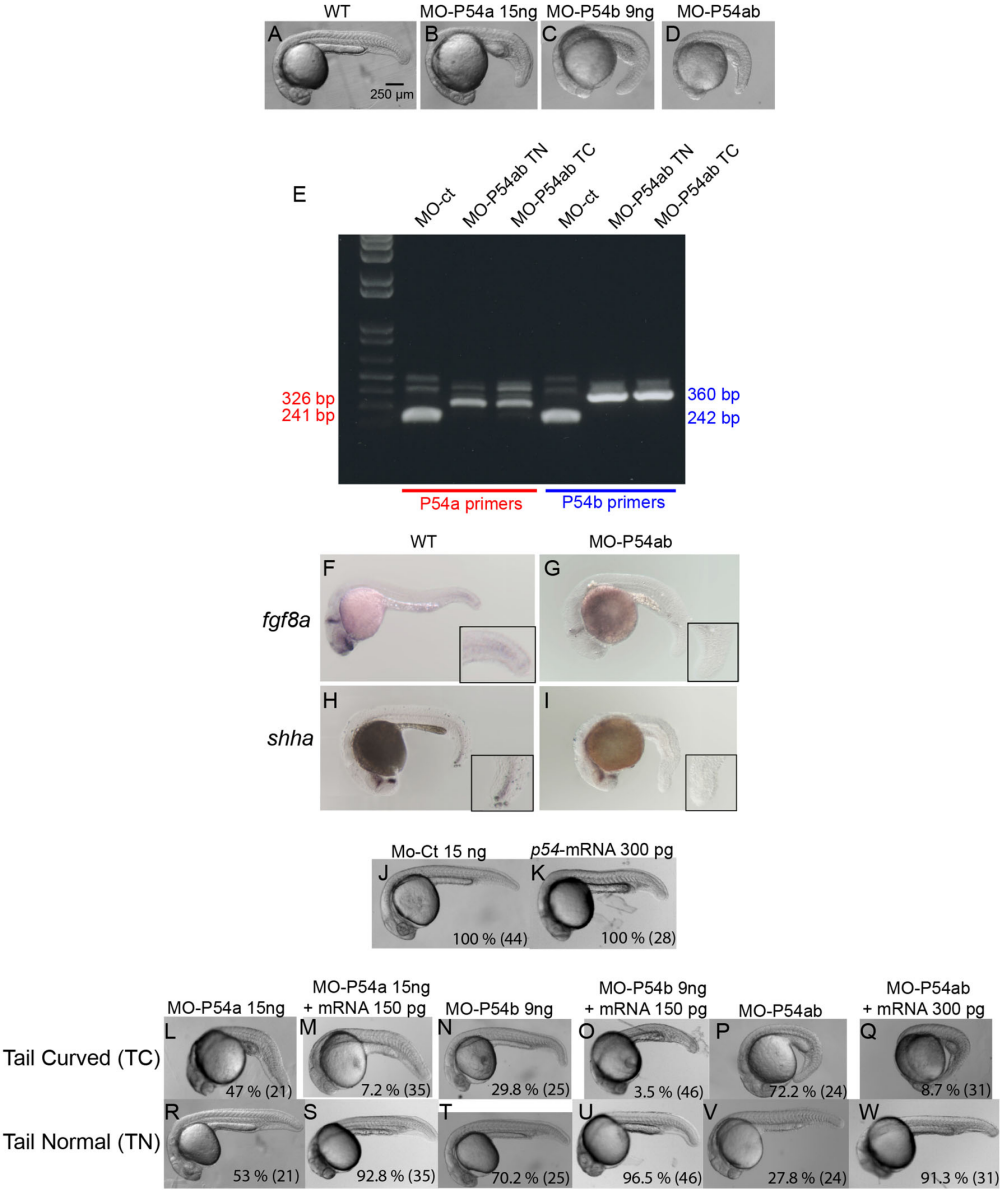
**Knockdown of P54a and/or P54b RNA helicases in zebrafish embryos.** (A–D) show the defects caused by MO-P54a, MO-P54b or MO-P54ab, which mostly affected posterior trunk structures and produce a bend at the end of the trunk (‘tail curved’). (E) Morpholino efficacy was validated by reverse transcription-PCR (RT-PCR). Using specific primers for *p54a* or *p54b* mRNA in samples from embryos micro-injected with MO-ct or MO-P54ab. Primers for amplifying *p54a* and *p54b*, under normal conditions, produced bands of 241 and 242 bp, respectively. However, in samples from MO-P54b embryos, bands of 326 or 360 bp were observed due to an intron insertion. (F–I) ISH showing the expression of *fgf8a* and *shha* in WT and MO-P54ab 24 hpf embryos. (J,K) There were no obvious defects in embryos micro-injected with 15 ng of MO-ct or 300 pg of *p54*-mRNA. (L–Q) The ‘tail curved’ phenotype produced by MO-P54a, MO-P54b or MO-P54ab was rescued if the morpholino was co-injected with *p54*-mRNA. (R–W) With every micro-injection, a proportion of embryos developed normally (‘tail normal’), but this proportion increased drastically upon rescue with *p54*-mRNA. (J–W) For each image, the percentage of embryos showing that phenotype is indicated, with the total number of embryos analyzed shown in parentheses. Control morpholino (MO-ct), *p54a* morpholino (MO-P54a), *p54b* morpholino (MO-P54b), mix of *p54a* and *p54b* morpholinos (MO-P54ab), ‘*in vitro*’ synthesized mRNA from *p54a* (*p54*-mRNA). ‘*in situ*’ hybridization (ISH), tail curved phenotype (TC), tail normal (TN).

When blocking P54a and/or P54b expression by splice-blocking morpholinos (MO-P54ab) we observed a 57% reduction in a 54 kDa band that is recognized by our anti-P54 serum in western blots from zebrafish protein extracts (Fig. S1F). Typical anti-P54 immunolabeling in cytoplasmic granules was also lost after morpholino treatment but rescued by the co-injection of morpholinos with *p54a* mRNA (Fig. S1G–I). As a consequence of the lack of *p54a* and/or *p54b* expression, we observed a consistent phenotype with more severe alterations in the posterior trunk than in anterior regions. The same phenotype was observed by knockdown with *p54a*, *p54b* or *p54ab* (a mix of MO-P54a and MO-P54b), except that morphological defects were more severe in *p54ab* double morphants, than in single (*p54a* or *p54b*) morphants (Fig. 5A–D). This phenotype consisted of several misshapen tissues, i.e., somites, notochord and blood vessels in posterior regions, with the posterior end of the trunk bending downward (we call this phenotype ‘tail curved’). In contrast, anterior tissues such as the brain, most anterior somites, anterior notochord, heart and anterior blood vessels appear normal or only mildly affected (Fig. 5A–D).

Pleiotropic zebrafish mutants are known to show defects first in anterior regions, since the rate of cell division is higher in the brain that in the rest of the body during early stages of development. However, this is not the case for *p54a* and *p54b* morphants, indicating that it is a specific phenotype. Splice-blocking morpholinos were efficient at disrupting *p54a* and *p54b* RNA maturation because we observed intron insertion events for both genes when microinjecting either MO-P54a or MO-P54a (data not shown) or when microinjecting a mix of the two morpholinos (Fig. 5E).

Two mRNAs known to be expressed in both the head and the posterior trunk (*fgf8a* and *shha*) were tested by *in situ* hybridization (ISH) in *p54ab* double morphants. We observed that, while anterior domains continued to express these particular genes, the posterior regions lost expression when both P54a and P54b were knocked down (Fig. 5F–I). Other markers, such as *krox20*, *bmp4*, *nog1*, *flh* and *ntl*, which are specific for anterior and/or posterior regions, were also tested by ISH, but no significant differences were found between wild type (WT) and morphants (Fig. S4). In order to determine whether this phenotype is in fact induced by the loss of P54a and/or P54b expression, we conducted a rescue experiment by co-injecting MO-P54a with *p54a*-mRNA, MO-P54b with *p54a*-mRNA or MO-P54ab with *p54a*-mRNA. The ‘tail curved’ phenotype that is usually present at rates of 47% (MO-P54a), 29.8% (MO-P54b) and 72% (MO-P54ab) was observed at only 7.2% (MO-P54a), 3.5% (MO-P54b) and 8.7% (MO-P54ab) in the presence of *p54a*-mRNA (Fig. 5J–W). As a control, embryos injected with the same amount of *p54a*-mRNA alone did not have phenotypic defects (Fig. 5K). These experiments suggest that knocking down P54a and P54b induces defects in the development of posterior trunk structures.

### P54 RNA helicases are required for resilience after stress treatment

**Fig. 6.**
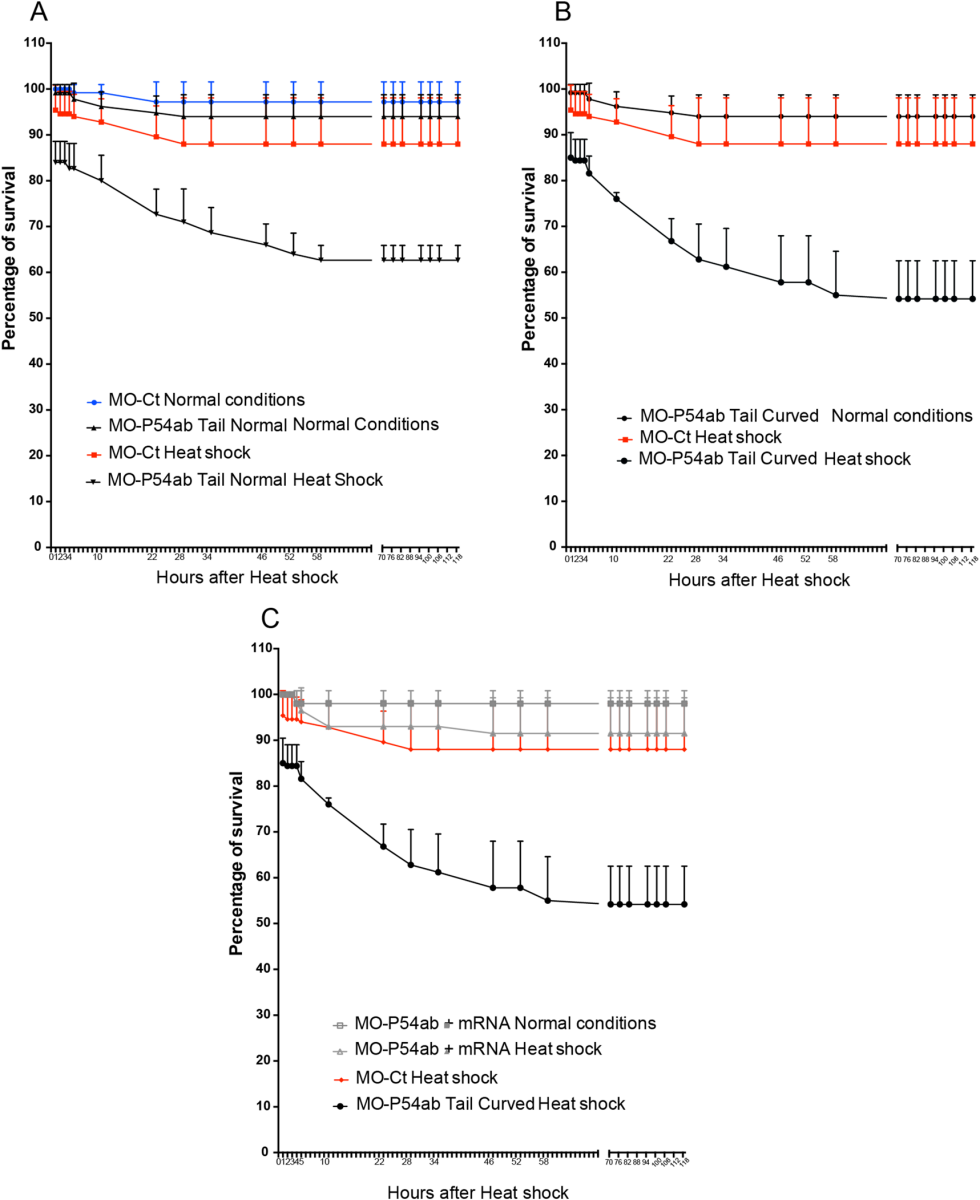
**Resilience after heat shock.** Comparison of survival rates between embryos maintained at 28°C (normal conditions) or exposed to heat-shock conditions at 37°C for 2 h (heat shock). Embryos were micro-injected with MO-Ct or MO-P54ab. The MO-P54ab micro-injected embryos were separated into two groups. (A) Morphants showing the tail normal phenotype and (B) morphants showing the tail curved phenotype. (C) Some embryos were co-injected with MO-P54ab plus P54a-mRNA in order to rescue the lack of P54. In each experiment the number of embryos used was between 30 and 40. For each category, the total number of embryos was counted every hour during the first 4 h of recovery and thereafter twice per day for five days. Error bars were made with standard deviation values.

It is known that translation is blocked by cellular stress such as heat shock and that while polysomes are disassembled, mRNAs stalled in translation become accumulated in stress granules. Stress granules are essential for conserving anabolic energy and preserving essential mRNAs later required for repairing stress-induced molecular damage (Bergkessel and Reese, 2004; Yamasaki and Anderson, 2008). We next tested whether the knockdown of P54 RNA helicases affects survival after heat-shock stress. Control and morphant embryos at 24 hpf were heat-shocked for 2 h at 37°C and then allowed to recover under normal conditions (at 28.5°C). For the purposes of this analysis, morphant embryos were classified into two groups, ‘tail curved’ (with phenotypic defects) and ‘tail normal’ (with no evident phenotypic defects). Data were collected hourly during the first 4 h of recovery for each group of embryos and thereafter twice per day for the 5 days after the stress treatment (Fig. 6).

When growing at normal temperature double-morphants MO-P5ab (co-injected with a mix of MO-P54a and MO-P54b) have higher rates of survival, approximately 95% after five days of development. In fact, they survive at a similar rate to zebrafish embryos micro-injected with a control morpholino (Fig. 6A,B). Heat shock alone reduced the survival rate of embryos micro-injected with control morpholino to approximately 85–88%. However, survival rates for double morphants (MO-P54ab) drop to 45% after heat shock. Phenotypic ‘tail curved’ P54ab morphants showed slightly lower survival rates (Fig. 6B) than ‘tail normal’ P54ab morphants (Fig. 6A). Furthermore, co-injection of MO-P54ab with *p54a* mRNA rescues embryos from lethality after heat shock to almost normal rates of survival (86%) (Fig. 6C). This result suggests that P54 RNA helicases are required for survival after heat shock treatment. Because we previously observed that knocking down P54 RNA helicases impairs the formation of stress granules (Fig. 3Q–R), we propose that P54ab morphants are less likely to recover after heat shock due to the loss of stress granules.

## DISCUSSION

We found that zebrafish possess two P54 Dead box RNA helicases coded by the genes *p54a* and *p54b* on chromosomes 18 and 16, respectively. Phylogenetic analysis show that these are co-orthologs of the tetrapod *p54* RNA helicase gene and likely appear in the teleost whole-genome duplication (Glasauer and Neuhauss, 2014). Both P54a and P54b are expressed in cytoplasmic granules in zebrafish embryos, consistent with previous results that P54 RNA helicases are components of P-bodies and stress granules (Bish et al., 2015; Minshall et al., 2009; Navarro et al., 2001; Paz-Gomez et al., 2014; Serman et al., 2007). Zebrafish P54 cytoplasmic granules resemble P-bodies under normal conditions and stress granules under heat-shock conditions. P54 RNA helicase and Dcp2 (mRNA decapping enzyme) are frequently used as markers for P-bodies (Ingelfinger et al., 2002; Minshall et al., 2009). In zebrafish, P-bodies labeled with anti-Dcp2 were similar in size to P54-containing granules. While P54a-mCherry and P54b-EGFP fusion proteins were often found in the same granules, we did not observe co-localization of P54 fluorescent reporters with Dcp2-positive granules, this was unexpected and may be explained by the existence of different classes of P-bodies, even in the same cell, as was proposed before (Parker and Sheth, 2007). The cytoplasmic granules containing P54 helicases in zebrafish do not resemble germ granules but are highly similar to stress granules.

Stress granules are reversible aggregates of RNA-binding proteins and translation initiation factors and contain untranslated mRNAs. These granules assemble in response to stress conditions and are typically larger than P-bodies (Anderson et al., 2015). We found that by treating zebrafish embryos with a heat shock the formation of large cytoplasmic granules was induced. We propose these are stress granules because (i) they were heat-induced, (ii) they were larger than P-bodies, and (iii) they were labeled with the anti-TIAL-1 antibody (a known marker for stress granules) (Kedersha et al., 1999; Moutaoufik et al., 2014). In normal conditions fusion proteins (like P54a-EGFP, P54a-EGFP or P54a-mCherry) were detected in cytoplasmic granules with an average diameter of 0.3 μm, while in heat shock conditions the same fusion proteins were found in cytoplasmic granules with an average size of 1 to 1.6 μm in diameter. Our measurements are equivalent to previous reports for P-bodies and stress granules, respectively (Eulalio et al., 2007; Thomas et al., 2009). However, we failed to observe co-localization between P54 fusion reporters and anti-TIAL-1-positive cytoplasmic granules. It is interesting that very often we detected P54 heat shock granules adjacent to TIAL-1 granules. A similar observation was recently made in zebrafish cells in culture, where anti-eIF3e antibody-labeled stress granules were found in close contact with FUS-GFP-containing granules and coincidently both types of granules were induced under stress conditions (Acosta et al., 2014). In our case, both P54 large granules and TIAL-1 granules only appeared after embryo heat exposure.

We also observed that P54 heat-induced large granules were located in the same cells and in close contact with smaller P54 granules. It is known that stress granules and P-bodies are both present in stress conditions and that they are frequently close to each other (Kedersha et al., 2005; Souquere et al., 2009; Wilczynska et al., 2005). At the same time it was proposed that specific proteins or mRNAs are exchanged between P-bodies and stress granules (Buchan et al., 2008; Stoecklin and Kedersha, 2013); however, formation of both types of granules was induced in stress conditions (Acosta et al., 2014). Our observations were carried out mainly in epithelial cells from the skin and muscle cells, since these were easier to image in the 24 hpf treated embryos (Fig. S2). Even though stress granules have been reported to be widely distributed in many cell types and do not seem to be cell specific, some RNA granules, like SX-bodies (Thomas and Boccaccio, 2016) were only detected in neurons, therefore further work will be required to determine if there are different types of stress granules and if some of these could be cell-specific. We observed that knocking down the expression of P54a or P54b with morpholinos prevented the formation of stress granules in heat­-shock conditions, as detected by the anti-TIAL-1 antibody. This effect could be rescued by co-injection of *p54a* mRNA, suggesting that P54 helicases were required for stress granule assembly in zebrafish. This observation is in agreement with reports where P54 is required for stress granule assembly (Mollet et al., 2008; Thomas et al., 2009). Intriguingly, some authors working with mammalian cells have found the opposite; that P54 RNA helicases are not essential for stress granule formation (Serman et al., 2007) even though they are required for P-body assembly (Andrei et al., 2005; Chu and Rana, 2006). One possibility is that there are different mechanisms for stress granules assembly between mammals and zebrafish.

Stress-induced repression of translation is widely accepted to be connected to the formation of stress granules (Lascarez-Lagunas et al., 2014; Mollet et al., 2008), and we found this to be true as well for P54a and P54b heat-induced granules in zebrafish embryos. In our experiments, P54 heat-induced granules and TIAL-1-labeled stress granules showed the same responses to the blockade of translation by cycloheximide or by puromycin. While cycloheximide prevents stress granule assembly and forces the disassembly of pre-formed stress granules due to mRNA stabilization into polysomes, puromycin enriches the pool of mRNAs in stress granules (Wilczynska et al., 2005). Because these antibiotics change the amount of mRNA available for stress granules, P54 granules formed during heat shock are the direct consequence of translation arrest. Therefore, if the size of the P54-containing granules is determined by the supply of mRNAs stalled in translation, then they act just as genuine stress granules.

In addition to the apparent loss of stress granules upon P54a or P54b knockdown, we observed defects in the development of the posterior trunk region in treated zebrafish embryos, specifically affecting posterior somites, whereas anterior tissues were not severely affected. The consistently worse phenotype in posterior regions was surprising because P54-stained granules (labeled by the anti-P54 serum) were broadly expressed in 24 hpf zebrafish embryos. The fact that we observed the same phenotype upon knocking down the expression of either P54a or P54b suggests that these two RNA helicases perform similar functions. This is not unexpected since all eight functional domains (Q, I, Ia, Ib, III, IV, V and VI) are highly conserved between the two genes and because it is not uncommon that duplicated genes in zebrafish have the same function. P54a and P54b fluorescent reporters were also observed to co-localize in the same cytoplasmic granules when co-expressed, suggesting then same function and same subcellular localization. Unfortunately, this could not be confirmed with our anti-P54 antibody since it could not differentiate between P54a and P54b. RT-PCR (reverse transcription PCR) analysis from whole embryos showed that both are expressed from early on and in the same developmental stages (data not shown). The fact that double morphants (co-injected with a mix of MO-P54a and MO-P54b) showed worse defects than single MO-P54a or MO-P54b morphants suggests functional redundancy as has been reported for other duplicate genes in zebrafish (Campos et al., 2012; Dougan et al., 2003). This is also supported by the fact that MO-P54b morphants and MO-P54ab double morphants are efficiently rescued by the micro-injection of *p54a*-mRNA.

Surprisingly, even in severely affected double-morphant embryos, most of the genes we assayed by *in situ* hybridization showed no disruption in expression patterns. However, in double morphants, the expression of both *shha* and *fgf8a* was completely lost in the posterior trunk but still observed in anterior regions. One of the main characteristics of P54 RNA helicase morphants is that posterior somites were clearly misshapen. The genes *shh* and *fgf8* participate in somite development (Boulet and Capecchi, 2012; Resende et al., 2010), raising the possibility that P54 RNA helicases or the cytoplasmic granules they form are somehow part of the *shh*- and/or *fgf8*-related mechanisms of somite development. Notochord underdevelopment impairs *shh* and *fgf8* expression, but the notochord marker *ntl* was indeed expressed in single and double morphants from P54 RNA helicases, showing that notochord formation is not impaired. We looked for a relationship between the function of stress granules and somite development in zebrafish and found that P97a and P97b, homologous proteins to the translation initiation factor eIF4G, are components of stress granules and both are required in zebrafish for mesoderm formation (Nousch et al., 2007). At the same time we found that the mammalian Disheveled (Dvl) protein, an effector of the Wnt signaling pathway, negatively regulates the assembly of stress granules (Sahoo et al., 2012) and it is known that the Wnt pathway is involved in zebrafish somite formation (Bajard et al., 2014).

Repression of translation is a general response to different stresses in many organisms, which involves many different molecular events and cellular mechanisms. During stress mRNAs stall in translation initiation and are transferred from polysomes to stress granules. At the same time, the selective translation of mRNAs encoding repair enzymes is initiated (Yamasaki and Anderson, 2008). It is known that the lack of some stress granule components affect cell survival after an induced stress; for example, the serine/threonine kinase RSK2 (Eisinger-Mathason et al., 2008) or the cytoplasmic deacetylase HDAC6 (Kwon et al., 2007). It is widely accepted that stress granules are required for a rapid recovery after stress (Anderson and Kedersha, 2006; Kedersha and Anderson, 2007) but for this to happen, it has been shown that the presence of P54 RNA helicase is important. For example, yeast do no reinitiate the cell cycle after an induced stress in the absence of their P54 homolog (known as DHH1) (Bergkessel and Reese, 2004).

We explored the resilience of zebrafish embryos with decreased levels of P54 RNA helicases and stress granules. In double MO-P54ab­-morphant embryos we found that the ability to recover from heat-shock stress is strongly impaired and only rescued by the addition of exogenous *p54a* mRNA. These data support the idea that P54 RNA helicases are essential for the formation of stress granules, and therefore for the resilience of organisms after heat shock. It has been observed that once the stress has ended mRNAs stored in stress granules move back to polysomes (Mollet et al., 2008); therefore, stress granules may prevent mRNA degradation during stress conditions (Kedersha et al., 2005). It is also possible that zebrafish P54 RNA helicases have a role in protecting mRNAs for degradation during the heat shock, and for that to happen a large amount of P54 is necessary in the cells. It has been estimated that mammalian cells in culture may contain as many as two million P54 molecules per cell (Ernoult-Lange et al., 2012). Since the number of mRNAs per cell has been calculated to be from 20,000 to 300,000 (Ernoult-Lange et al., 2012; von der Haar, 2008) there seems to be at least a sevenfold molar excess of P54 RNA helicases with respect to mRNAs.

P54 RNA helicases also interact with microtubules (Rajgor et al., 2014) so it is also possible that these RNA helicase participate in mRNA transport during stress. It has also been proposed that stress granules prevent apoptosis by sequestration of apoptosis-inducing factors like RACK1 (Buchan and Parker, 2009). Stress granules also inhibit apoptosis by reducing the production of reactive oxygen species (Takahashi et al., 2013). In our experiments zebrafish embryos with reduced levels of P54 RNA helicases and stress granules could be affected by mRNA mislocalization and degradation, as well as increased apoptosis. In conclusion, stress granules and P54 RNA helicases may be important as part of mechanisms for recovery after stress conditions.

## MATERIALS AND METHODS

### Zebrafish strains and growth conditions

All procedures performed with animals were approved by the Office of Laboratory Animal Welfare (OLAW) of the United States National Institutes of Health (NIH), approval #A5281-01. Wild-type zebrafish (*Danio rerio*) embryos were obtained from natural crosses of our Tab-WIK strain, a cross from the strains TAB-14 and WIK obtained from the Zebrafish International Resource Center (ZIRC). Adult zebrafish were maintained in a recirculation system (Aquatic Habitats) with a constant pH, a 28°C temperature and a light:dark cycle of 10:14 h (Trevarrow, 2004). Some experiments were carried out with the wild-type strain AB that was maintained in the zebrafish facility at the Genetics-Biotechnology Center at the University of Wisconsin-Madison. Freshly fertilized embryos were incubated at 28.5°C in Embryo Medium (5 mM NaCl, 0.17 mM KCl, 0.33 mM CaCl_2_, 0.33 mM MgSO_4_) (Westerfield, 1993).

### Bioinformatics analysis

We used the human RCK amino acid sequence (NM_001257191) as bait in BLAST searches against the zebrafish genome sequence using Ensembl databases. We obtained two annotated sequences, ENSDARP00000081816.5 (Chromosome 18:43.87 Mb) and ENSDARP00000129311.1 (Chromosome 16:25.76 Mb). These proteins were identified as P54 RNA DEAD box helicases after multiple alignment and phylogenetic tree construction. P54 RNA helicase sequences from zebrafish and other organisms (human RCK, mouse DDX6, *C elegans* CGH_1, *Drosophila* Me31B, *Xenopus* DDX6, medaka DDX6_A and medaka DDX6_B) were aligned using ClustalW2. Pro-Test 3.2 was used to calculate the substitution model, and RAxML was used to generate the phylogenetic tree (Stamatakis et al., 2008) and to determine the identity between homologous sequences. The phylogenetic tree outgroup consisted of the homologous proteins zebrafish eIF4A_1A, zebrafish eIF4A_1B, human eIF4A and *C. elegans* eIF4A.

### Anti-P54 serum testing

To investigate the expression of P54 RNA helicases in zebrafish during development, we developed a rabbit antiserum against the synthetic peptide YDDRFNLKGIEEQL derived from a C-terminal sequence from zebrafish P54a. This antibody may also recognize P54b because the corresponding sequence (SEDRFNLKGIEDQL) is highly conserved as shown in a protein alignment (Fig. S1B), where 13 out of 14 residues were either identical or conserved substitutions. In western blot analysis from whole embryo extracts, both P54 proteins were indistinguishable, with calculated molecular weights of 53.9 and 54.3 for P54a and P54b, respectively. The anti-P54 serum identified a broad band of approximately 54 kDa, (Fig. S1A) that was not labeled by the pre-immune serum. The relative density for each P54 and tubulin band, in the western blot experiment, was calculated used ImageJ (Schneider et al., 2012). Relative density for P54 bands was normalized against its corresponding relative density tubulin loading control band. When tested by whole-mount immunostaining in 24 hpf embryos, the P54 serum labeled cytoplasmic granules in most cells, and again, the pre-immune serum did not show any specific labeling (Fig. S1C,D).

### Morpholino knock down

Splice-blocking morpholinos were designed to block the donor splice site at the exon 1-intron 1 boundary in both *p54a* and *p54b* (Bill et al., 2009) (see (Table S1). After titration by RT-PCR (reverse transcription-PCR), micro-injection of 15 ng of MO-P54a or 9 ng of MO-P54b was found to induce the complete loss of normal *p54a* and *p54b* mRNAs, producing insertions of intron 1 in both cases. The primers for testing the expression of *p54a* and *p54b* mRNAs (cDNAs) using RT-PCR were based on exon 1 and exon 3 sequences (see Table S1). Micro-injection of a mix of 15 ng of MO-P54a and 9 ng of MO-P54b produced the same results by RT-PCR and a phenotype (discussed in the Results section) similar to those produced by separate MO-P54a and MO-P54b micro-injections. Western blot analysis of double morphants (MO-P54a+MO-P54b) showed a 57% decrease in the expression of the 54 kDa band as identified by the anti-P54 serum and calculated as relative densities using ImageJ (Fig. S1E,F). In whole-mount immunostaining, we observed the loss of the typical anti-P54 labeling in double MO-P54a+MO-P54b morphants. Such labeling of cytoplasmic granules was rescued by co-injection of the mix MO-P54a+MO-P54b+*p54*-mRNA. For western blotting, protein extracts of 12 WT or morphant embryos at 24 hpf were run in each lane of a polyacrylamide gel. After transfer, rabbit polyclonal serum against P54 was used at a dilution of 1:2000. The loading control, rabbit-anti-α-tubulin (Abcam, ab15246) was used at a dilution of 1:500. The secondary antibody, goat polyclonal anti-rabbit IgG-HRP (Abcam, ab97051) was used at a dilution of 1:10,000. Micro-injections were carried out using air pulses at 25 psi with a MINJ-1 micro-injector (Tritech Research) using pulled glass needles (P-1000 Sutter Instruments) with 1 mm outer diameter and 0.58 mm inner diameter, positioned with a three-axis Narishige micromanipulator. RT-PCR was carried out using TrIzol-based total RNA extraction of 24 hpf WT or morphant zebrafish embryos, followed by first-strand cDNA synthesis using SuperScript^®^ III Reverse Transcriptase (Thermo Fischer) with oligo dT primers according to the manufacturer's recommendations. PCR was carried out using primers from; actin (as a control), *p54a*-mRNA and *p54b*-mRNA (see Table S1).

### Zebrafish whole-mount immunostaining

Zebrafish embryos at different stages of development were fixed overnight in cold 4% paraformaldehyde-PBS (PFA). Embryos were then dehydrated with methanol for storage at −20°C and rehydrated, washed in PBST [PBS+0.1% Tween 20], permeabilized with cold acetone (20 s), blocked with goat serum for at least 1 h at room temperature. Thereafter, they were incubated with rabbit anti-P54 serum (1:2000), rabbit pre-immune serum, rabbit anti-Dcp2 (Novus Biologicals, NBP2-16109; 1:2000), rabbit anti-TIAL-1 (Novus Biologicals ,NBP1-79932; 1:2000), rabbit anti-phosphorylated non-muscle myosin (NMII-p) (Cell Signaling, 3671) or rabbit anti-DDX4 (anti-Vasa) (Abcam, ab13840), these were diluted in PBST and incubated overnight at 4°C. Labeling was detected using secondary antibody (1:1000) goat anti-rabbit IgG conjugated with Alexa Fluor 488 (Jackson ImmunoResearch, 111-545-003), and nuclei were counterstained with DAPI (4′,6-diamidino-2-phenylindole).

### Fusion reporter proteins expression and RNA rescue assay

Full open reading frames (ORFs) for both *p54a* and *p54b* were obtained by RT-PCR (see primers sequences in Table S1), digested with *Bam*HI and *Cla*I enzymes and cloned in pCS2 or pCS2+8CmCherry for expression. For EGPF expression both genes were cloned in the Gateway cloning system (Thermo Fischer) entry vector pDONR 221 and further recombined into the expression vector pCDNA 6.2/EmGFP-DEST. In all cases fusion fluorescent reporter proteins were placed at the C-terminal end of P54 proteins. For expression 75 pg of each plasmid were micro-injected as mentioned above. For rescue experiments capped *p54*-mRNA was *in vitro* synthesized using the mMessage mMachine SP6 kit (Ambion) and 300 pg were micro-injected in fish embryos.

### Whole mount *in situ* hybridization

Plasmids were linearized before the *in vitro* transcription using T7, T3 or SP6 RNA polymerases. Digoxigenin-labeled antisense probes were used to detect mRNA expression of *fgf8a*, *shha*, *krox20*, *bmp4*, *nog1*, *flh* and *ntl*. An anti-digoxigenin antibody (Roche Life Sciences, 11093274910) conjugated with alkaline phosphatase was used to detect the hybridization pattern in 24 hpf zebrafish embryos pre-fixed with cold 4% PFA and permeabilized by a short treatment with proteinase K. All plasmids containing fragments (approximately 400 to 1000 bp) of the tested genes were kindly provided by Professor Isaac Skrome at the University of Miami, except for *nog1* and *flh*, which were cloned by us (Table S1).

### Visualizing zebrafish embryos

Treated embryos were mounted in 2% agarose in embryo medium, and images were obtained with a confocal microscope (FV10i Olympus). Some images were obtained with an epifluorescence microscope (Axioimager Zeiss) equipped with an AxioCam MRc camera and the ZEN image capture software (Zeiss). All images were processed with Illustrator CS6 software (Adobe). Embryos treated for ISH were photographed with a Sony Cybershot camera DSC-H20 attached to a Stereoscopic microscope SMZ-645 (Nikon) by means of a MM99 adaptor (Martin Microscopes Company). In embryos from immunostaining or expression experiments two images were obtained, one in a low amplification (10×) and one in high amplification (60×). The low amplification image was used as a reference of the body region were the image was obtained (see insets in Fig. 2D,G–H,K and Fig. 3A–D,Q–S). All images from 24 hpf embryos were obtained from anterior regions of the trunk. The cells more often photographed were epithelial cells from the skin and in some cases muscle cells. We were able to identify the cell type using DIC (differential interference contrast) illumination (Fig. S2), aided by the cell shape (GFP expression) or nuclei form (DAPI stained), see for example epithelial cells in (Fig. S2E,G,I) versus muscle cells in (Fig. S2H,Q). Calculations for the average diameter of P54a-EGFP and P54b-EGFP cytoplasmic granules were made using ImageJ (Schneider et al., 2012). First we calculated the area (μm^2^) for all the granules in five cells in each condition (including normal temperature and heat shock) and the averaged area was converted to diameter, assuming each granule to be a circle (Fig. S3).

### Heat-shock assays and survival assays

Synchronized zebrafish embryos were incubated in Petri dishes at 28.5°C until 24 hpf and were subsequently transferred to an incubator for heat shock (30–40 embryos per plate) and incubated at 37°C for 30 min to 2 h. For immunostaining experiments, heat-shocked embryos were fixed in cold 4% PFA immediately after heat shock; for survival assays fish embryos were moved to 28.5°C after the heat shock, and the number of surviving embryos was registered during the following 4 days. For survival experiments, data were obtained from multiple experiments, and the total survival percentage was plotted. Three experiments were conducted for morphant animals, and three experiments were conducted for rescued animals. Analysis was carried out using Prism 6 software (GraphPad).

### Cycloheximide and puromycin treatments

Inhibition of translation was achieved under normal or heat-shock conditions using 30 μg/ml cycloheximide (Roche Diagnostics) or 80 μg/ml puromycin (Sigma) in Embryo Medium for 2 h. After treatment, embryos were immediately fixed in cold 4% PFA.
